# Exosomal ncRNAs in liquid biopsy: a new paradigm for early cancer diagnosis and monitoring

**DOI:** 10.3389/fonc.2025.1615433

**Published:** 2025-07-09

**Authors:** Muhammad Usman Ghani, Liang Du, Abdulkareem Qasem Moqbel, Erhu Zhao, Hongjuan Cui, Liqun Yang, Xiaoxue Ke

**Affiliations:** ^1^ Medical Research Institute, State Key Laboratory of Resource Insects, Southwest University, Chongqing, China; ^2^ College of Biological Sciences and Biotechnology, Beijing Forestry University, Beijing, China

**Keywords:** exosomal ncRNA, miRNAs, lncRNAs, circRNAs, biomarkers, liquid biopsies, non-invasive

## Abstract

Cancer’s aggressive nature and delayed diagnoses often result in poor prognoses and limited treatment outcomes. Early detection, personalized treatments, and effective monitoring are essential for improving cancer management. Traditional tumor biomarkers, such as beta-2 microglobulin and Carcinoembryonic Antigen (CEA), are often yield inaccurate and inconclusive results. Recently, exosomal cargoes, especially non-coding RNAs (ncRNAs) such as microRNAs (miRNAs), long non-coding RNAs (lncRNAs), and circular RNAs (circRNAs), have gained attention as promising tools for the early, non-invasive detection of cancer. For instance, serum exosomal long ncRNA FOXD2-AS1 has demonstrated promising diagnostic potential in colorectal cancer (CRC), achieving an overall AUC of 0.736 across all patients and an improved AUC of 0.758 specifically for early-stage CRC, highlighting its effectiveness as a stage-specific biomarker for early detection and clinical assessment. Similarly, exosomal lncRNA-GC1 has effectively distinguished gastric cancer patients from controls and related conditions, with AUCs exceeding 0.86, thereby outperforming traditional markers such as CA 72-4, CEA, and CA19-9, which all scored below 0.79. Despite their great potential, the clinical application exosomal ncRNAs remains limited. This review highlights recent advancements in exosomal ncRNA research and their potential as diagnostic markers, addressing both the opportunities and challenges for clinical implementation.

## Graphical Abstract

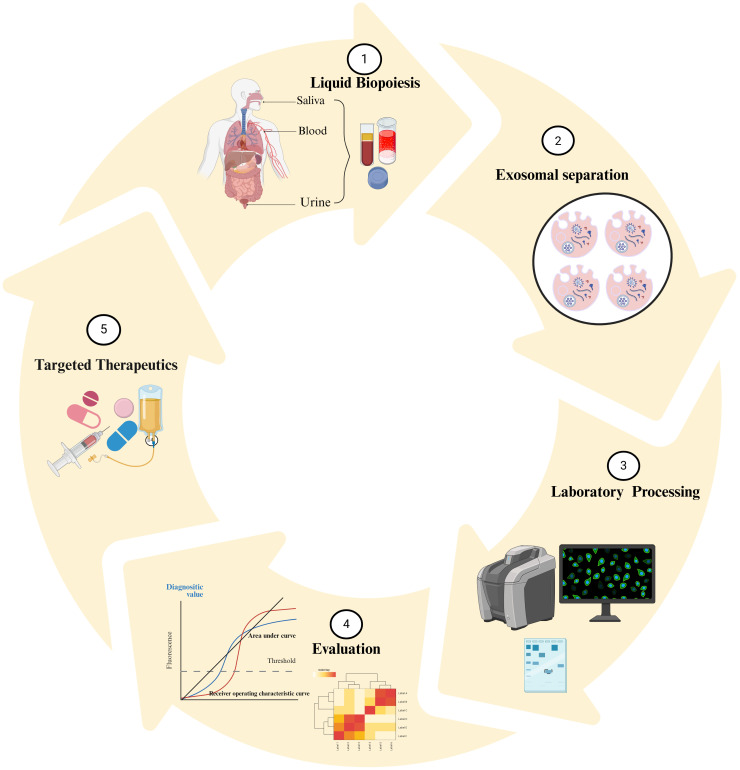

## 1 Introduction

Cancer is a multifaceted and formidable disease that presents a significant risk to global health. It occurs when a cell’s genetic material is mutated, leading to abnormal cell division and unresponsiveness to the body’s natural defense system. Despite significant research advancements over recent decades, it remains the second most commonly diagnosed disease and the fifth leading cause of death among non-infectious diseases worldwide (1). Prompt diagnosis is crucial, as it significantly improves treatment, prognosis, and overall survival (OS). Conventional approaches for early diagnosis include biopsy (2), ultrasound imaging, computed tomography (CT) scan (3), magnetic resonance imaging (MRI) (4), and markers in body fluids such as saliva, sweat, lymph, blood and urine (5).

Physical screening techniques pose significant challenges in cancer detection, such as high costs and the likelihood of false diagnosis. For example, profiling early-stage malignancies, such as CRC, may encounter challenges such as invasiveness, pain, and potential bleeding, which could cause patients to hesitate to undergo frequent examinations. Additionally, distinguishing between colonic ischemia, ongoing clostridium difficile infection, benign polyps, and malignant tumors increases the risk of misdiagnosis (6). In these circumstances, liquid biopsy presents a promising non-invasive method. By investigating markers such as circulating free RNA (cfRNA) (7), circulating tumor cells (CTCs), tumor-derived vesicles (TDVs) (8), and exosomal ncRNA (9) found within body fluids can enhance the ability to identify and evaluate these oncogenic substances in the blood circulation, and offers a promising approach for early detection.

Exosomes are small extracellular vesicles (EVs) that were initially discovered in ovine reticulocytes in 1983 (10). At first, they were considered as cellular waste, but later investigations revealed that they originate from majority of cell subtypes and are found in enriched culture medium and various body secretions (11, 12). They play a pivotal role in cell-to-cell interactions by targeting specific receptors on recipient cells, encapsulate and transport biomolecules such as enzymes, chemokines, cytokines, proteins, and ncRNAs (12).

The ncRNAs, formerly thought to be transcriptional by-products and considered as ‘junk RNAs,’ are now recognized as essential regulators of biological functions. The differential expression of certain ncRNAs such as miR-335, miR-383, miR-27a/b, and miR-376c, in exosomes from patients with HER2-positive and triple-negative breast cancer (TNBC), compared to healthy individuals, highlights the importance of exosomal ncRNAs in cancer (13–15). Additionally, the packaging of lncRNAs such as MALAT1, PCGEM1, and FAL1 in exosomes affects cancer development and metastasis (16, 17). Remarkable progress has been made in understanding the role of ncRNAs in cancer; however, an important gap remains in research regarding their specific prognostic potential. Most current studies focus on the mechanistic role of ncRNAs, highlighting their involvement in tumorigenesis and metastasis. However, the potential of exosomal ncRNA cargo in exosomes to serve as reliable markers for predicting clinical outcomes—such as survival, recurrence, and treatment response—has not been explored. This article aims to analyze the latest research developments and technological breakthroughs in this field, presenting exosomal ncRNAs as novel, non-invasive, highly specific, and sensitive prognostic markers for various cancers. It also addresses key challenges for clinical implementation, including standardization, sensitivity, specificity, and the need for large-scale validation studies. Additionally, we present ongoing clinical investigations that provide a solid foundation for the future exploration of exosomal ncRNAs as promising tools in both diagnostics and therapeutic applications.

## 2 Exosome’s biosynthesis, principal components, and distinct cellular role

Exosomes are small lipid bilayer vesicles that originate from endosomes produced by nearly all cell types in the body. The transformation of early endosomes into late endosomes is significantly enhanced within cellular compartments, particularly the Golgi apparatus. These late endosome compartments, also known as multivesicular bodies (MVBs), are formed through two evolving processes: the ESCRT-dependent pathway and the atypical ESCRT-independent pathway. Both pathways involve membrane invagination, leading to the formation of intraluminal vesicles (ILVs) (18). The first pathways encompass more than thirty proteins arranged into 4 families (ESCRT 0, to III) (19, 20). ESCRT-independent pathways utilize the ceramide-mediated trafficking module, demonstrating TSPAN6, CD81, and CD151 (21). Once generated, ILVs are either destroyed in the lysosome following fusion with the late endosome or, in certain circumstances, released as exosomes, a kind of extracellular vesicle (EVs). According to the International Society for Extracellular Vesicles (ISEV) in 2018 criteria, the standard name of EVs is based on the biological composition, physical properties (structural size and structural density), and precursor cell (22).

**Figure 1:**
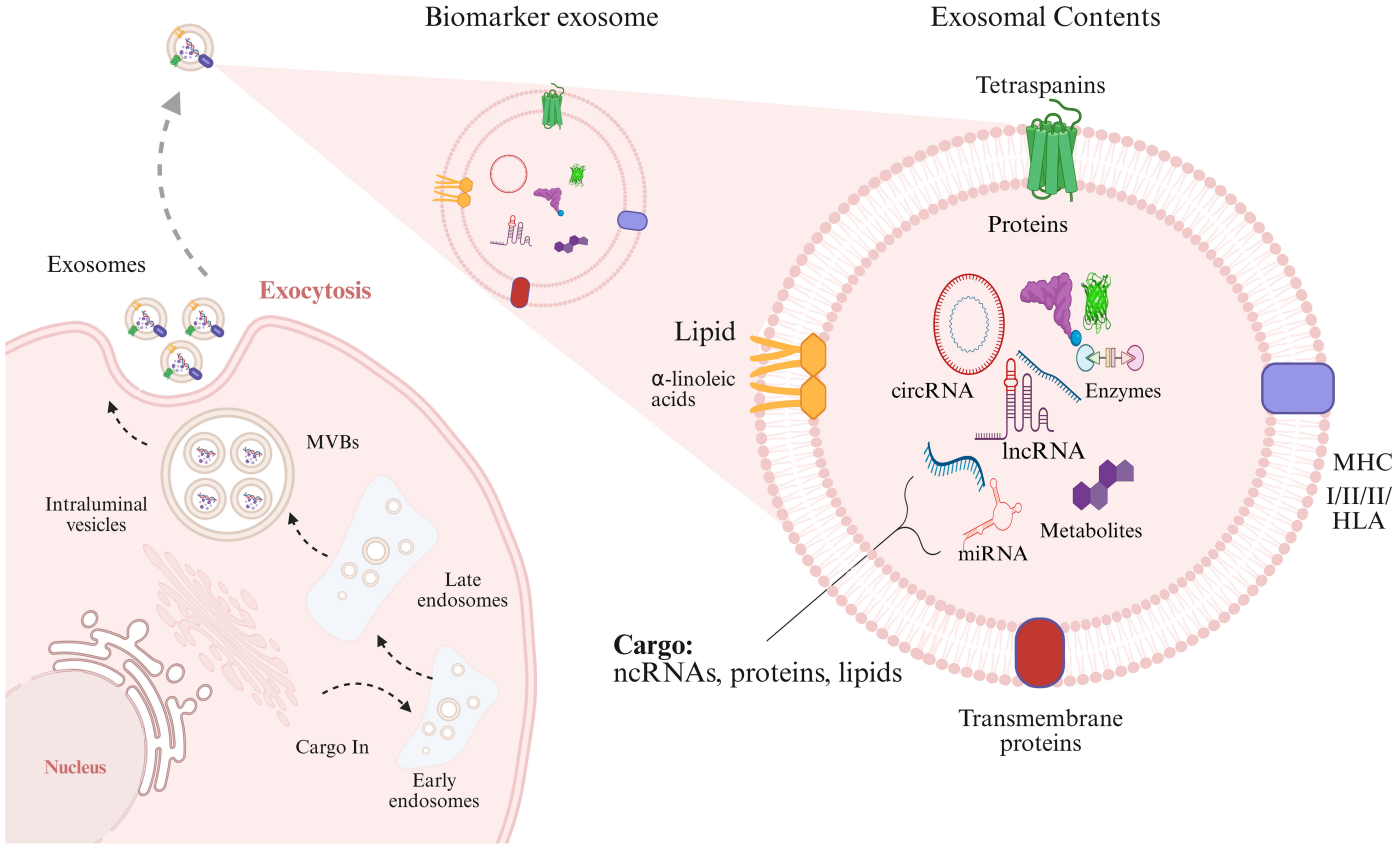
The first phase of the exosome biogenesis is endocytosis, contributing to early endosomes formation. Endosomes and specific cargoes are subsequently wrapped in Multivesicular Bodies (MVBs). At final step, MVBs attach with the membranous structure and exosomes are exported to extracellular matrix. The contents of exosomes (enzymes, proteins, DNA, ncRNAs) are transferred to target cells by direct fusion of membranes, receptor interactions, and endocytic process.

Exosomes contain a range of biologically active compounds, such as proteins, fatty acids, enzymes, DNA, and ncRNAs (miRNA, lncRNAs, circRNAs, snRNAs, snoRNAs, piRNAs, and tRFs). Additionally, exosomes carry molecules related to the major histocompatibility complex (MHCI & II), tetraspanins (TSPAN1, TSPAN6, CD81, and CD151), adhesion molecules (ICAMs, VCAMs), and membrane proteins like integrins and cadherins. Recent studies suggest that cellular stress, such as oxidative damage and hypoxic conditions within the tumor microenvironment trigger a marked increase in exosome production. This enhancement is particularly pronounced in tumor cells, which generate exosomes at a significantly faster rate than normal cells, resulting in considerably higher concentrations of exosomes in the bodily fluids of cancer patients. Additionally, factors such as overexpression of p53, elevated levels of heparanase, and increased Rab GTPase enzymatic activity further stimulate exosome secretion. These conditions not only increase the number of exosomes but also change their content, making them valuable for non-invasive diagnostic applications. **Figure 1** illustrates the biogenesis of exosomes and their molecular cargo.

## 3 Insights into exosomal ncRNAs

In the have previous decade, breakthrough progress in next generation sequencing and genome annotation strategies has unveiled numerous classifications of ncRNAs. They account for 98% of human genome transcripts and are classified by length into two categories: small non-coding RNAs (sncRNAs), which measure fewer than 200 nucleotides (nts), and long non-coding RNAs (lncRNAs), which exceed 200 nts (23–25). These ncRNAs are classified into several types based on their compositional and functional properties, including ribosomal RNAs (rRNAs), microRNAs (miRNAs), long non-coding RNAs (lncRNAs), circular RNAs (circRNAs), piwi-interacting RNAs (piRNAs), small nuclear RNAs (snRNAs), and small nucleolar RNAs. Recent evidence has shown that exosomes can encapsulate and transport a variety of ncRNAs, particularly miRNAs, lncRNAs, and circRNAs, and the aberrant behavior of these cargoes plays a crucial role in signaling pathways involved in cancer progression and metastasis. MicroRNAs such as miR-34a and miR-146a target PI3K and its downstream components, such as mTOR and GSK-3β, promoting cellular differentiation, proliferation, and invasion, which contribute to cancer development (26). Exosomal miR-96 promotes tumor growth, cellular invasion, angiogenesis, metastasis in lung cancer by targeting LMO7 (27). Exosomal miR-216b-5p from gemcitabine-resistant H1650 cells is transferred to specific cells, promoting cellular differentiation, multiplication, and infiltration by targeting the SOCS3 (28). Exosomal miR-21 from human bronchial epithelial (HBE) cells promotes neovascularization by stimulating STAT3 and inducing VEGF expression (29). Additionally, serum exosomal miR-92b-5p levels are associated with proangiogenic signaling in lungs cancer. Non-Small Cell Lung Cancer (NSCLC) enhance angiogenesis by inhibiting the cell-cell adhesion protein ZO-1 via exosomal miR-23a (30). They also release exosomal miR-214, which can promote angiogenesis and accelerate lung cancer growth.

**Figure 2:**
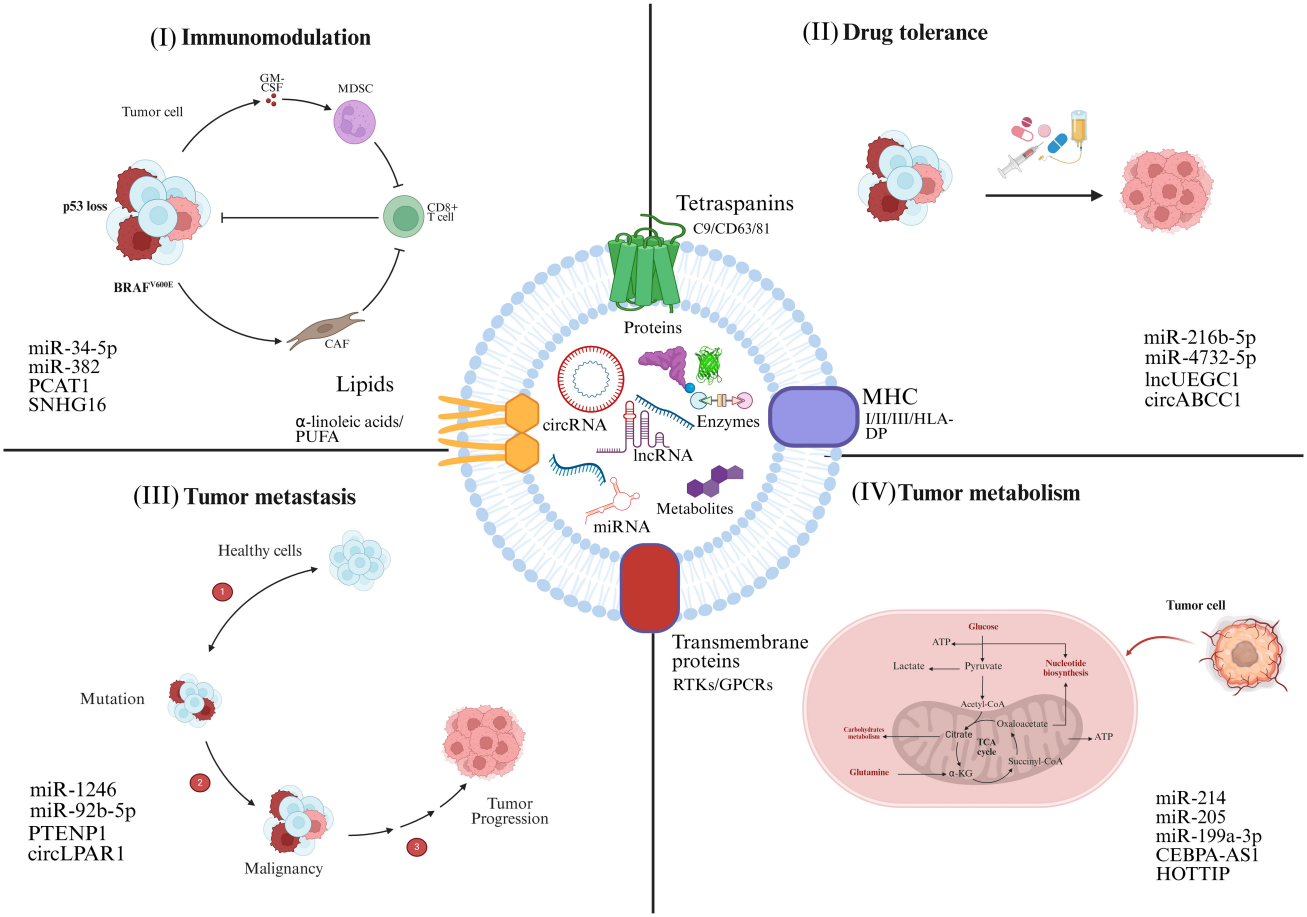
Tumor cells secrete exosomes that act as vehicles for the transport of ncRNAs. ncRNAs exert a wide range of effects on recipient cells, significantly influencing tumor progression. **(I)** Exosomal ncRNAs activate oncogenic signaling pathways, upregulate cell cycle-related genes, and inhibit tumor suppressor genes, thereby promoting rapid and unchecked tumor cell growth. **(II)** ncRNAs modulate the expression of drug-efflux pumps, and reprogramming epigenetic markers, contribute to the pharmacoresistant. **(III)** Reshape the immune microenvironment by regulating cytokine production, altering antigen presentation, and impairing the activity of cytotoxic T lymphocytes (CTLs), natural killer cells, and macrophages. **(IV)** Influence biochemical cycles, for example glycolysis, lipid metabolism, and oxidative phosphorylation, to adapt the metabolic profile of cancer cells for enhanced energy production and survival under nutrient-deprived conditions. The corresponding exosomal ncRNAs involved in these processes are highlighted.

Similar characteristics have also been observed in lncRNAs, which interact with transcriptional regulators, promotor sequences of genes, and allelic site to influence signal transduction cascades, thereby exerting either oncogenic or tumor-suppressive effects. Interestingly, most lncRNAs can be easily traced in different body fluids, making it a promising marker for early cancer diagnosis. For instance (31), observed noticed that the levels of SAP30L-AS1 in exosomes isolated from serum were upregulated in benign prostatic hyperplasia (BPH), while higher SChLAP1 has been recorded in prostate cancer (PC) as compared to BPH and normal control (32). Accordingly, the prostate-specific antigen (PSA) was used to detect the levels of SAP30L-AS1 and SChLAP1, which showed clear distinction of their levels in benign and malignant cancers (33). Likewise, the upregulated serum exosomal XIST levels in patients with recurrent tiple- negative Breast Cancer (TNBC), compared to those with non-recurring or post-operative TNBC, signify its potential as a diagnostic marker for TNBC (34, 35). CircRNAs can also serve as miRNA sponges, binding to miRNAs and reducing their regulatory impact on mRNAs during transcription. **Figure 2** comprehensively demonstrates the significant role of exosomal ncRNAs in a broad spectrum of biological processes associated with cancer, including tumor progression, metastasis, immune evasion, and therapy resistance.

Considering the critical role of ncRNAs in cancer onset, invasion, and metastasis as part of the complex cargo within exosomes, we examined recent advancements in identifying exosomal ncRNAs as potential non-invasive molecular markers for pre-onset cancer detection. This analysis aims to enhance our understanding of their potential as both diagnostic tools and therapeutic targets. We compare the specificity, sensitivity, and AUC score of exosomal ncRNAs to other cancer markers, highlighting their potential for preemptive diagnostic tool.

### 3.1 Exosomes-derived microRNA

MicroRNAs (miRNAs) are small but important subset of small ncRNAs. These are single-stranded molecules, typically around 22 nts in length, that influence transcriptional activity by binding to open reading frames (ORF) or the 3′-untranslated region (UTR) of the target mRNA (36, 37). So far, 2,654 mature miRNAs have been recognized in humans (38). These miRNAs are involved in cellular functions under both normal and pathological conditions, including cells growth, differentiation, apoptosis, and metastasis.

Compelling evidence shows that exosomal miRNAs play an essential in driving tumor heterogeneity, promoting metastatic potential, and influencing prognosis by interacting with mRNA and negatively regulating its expression. For example, a significant increase in serum exosomal miR-22 was observed in patients with Oral Squamous Cells Carcinoma (OSCC), correlating with Stage III/IV tumors, lymphoid metastasis, and cellular inflammation (39). miR-22 is not only exclusive to OSCC; it may also serve as a marker in various malignancies, including gastric, lung, colorectal, and ovarian cancers (OC) (40–42). A panel of circulatory miRNA, comprising four extracellular miRNAs—miR-205, miR-193a-3p, miR-335, and miR-4732-5p—and seven exosomal miRNA markers, including miR-26a, miR-223, miR-429, miR-1229, miR-216b-5p, miR-1246, and miR-217-5p, demonstrated robust proficiency with an AUC score of 0.92, accuracy of 93%, a PPV of 96%, a sensitivity of 93%, and a specificity of 96% in screening the pancreatic ductal adenocarcinoma (PDAC) (43, 44). Several miRNAs, including miR-26, miR-122, and miR-150, have been identified as promising blood-based markers for the non-invasive clinical assessment of cholangiocarcinoma (45).

A study utilizing combinations of exosomal miRNAs, such as miR-21-5p and miR-24-5p, for lung cancer diagnosis has proven highly effective in differentiating NSCLC from controls, during the early phase. Furthermore, elevated level of exosomal miRNA such as miR-222 and miR-7797 in lung adenocarcinoma (LAC) patients were linked to lymphatic tissues metastasis and tumor severity, while exosomal miR-126 proved effective in distinguishing clinically normal control from early-phase NSCLC patients (45). As a diagnostic marker for NSCLC, a panel of serum-derived four miRNAs, comprising miR-205-5p, miR-9-3p, miR-1269a, and miR-210-5p, achieved AUCs of 0.914 in the trainee cohort and 0.877 in the confirmatory cohort (46, 47). Remarkably, suppression of miR-1269a and miR-205-5p inhibited tumor growth, invasion, and angiogenesis by targeting the FOXO1 gene, underscoring their diagnostic utility (48). In another study, plasma exosomal miRNAs levels were examined in three different groups including lung adenocarcinoma patients, healthy smokers, and those with pulmonary granuloma. The study identified that miRNAs, such as miR-200b-4p, miR-379, miR-192a-3p, and miR-139-5p, could differentiate lung adenocarcinoma from pulmonary granuloma, and the normal control group (49).

These miRNAs not only indicate the presence of cancer but also correlate with the disease stage and prognosis (50). have demonstrated that exosomes from MDA-MB-231 Breast cancer (BC) cell line exhibited upregulation of miR-210, which stimulating angiogenesis and cerebral metastasis in BC individuals. Survival outcomes are typically poor in patients with BC cerebral metastases exhibiting elevated miR-210 level. Interestingly (51), findings have highlighted that aggressive metastatic cell can transfer their potential to non-metastatic tumor cells through exosomal miRNAs. Exosomes expressing miR-200a released by BC cells were shown to transmit metastatic capabilities to non-metastatic cells, as established in humanized xenograft mice models. Exosomes can transport miR-770 and miR-105, regulating BC cell migration and metastasis (52, 53). miR-7641 has also been identified as non-invasive marker and a viable therapeutic target for breast cancer (54, 55). Higher levels of serum exosomal miR-373 have been associated with TNBC and may serve as valuable prognostic marker (56). BC patients exhibited elevated levels of exosomes containing miR-1246 and miR-21 in their plasma compared to normal controls (57, 58). Additionally, miR-155 acts as an oncogenic signal transmitted through secreted exosomes, facilitating intercellular communication and enhancing the aggressiveness of breast cancer (59, 60). It has been demonstrated that major changes in the exosomal content and miRNA levels are observed when comparing Lung’s adenocarcinoma (LUAD) patients to healthy controls. A notable degree of similarity was observed between the miRNA profiles derived from plasma exosomes and those originating from tumors. These findings suggest that exosomal miRNAs could play a pivotal role in the early detection of LUAD, although further research is needed to explore their broader diagnostic and prognostic implications (61). Exosomal miRNAs not only show promise as biomarkers for early-stage tumor detection, but also as predictive indicators for monitoring tumor behavior, treatment response, and the potential for metastasis. Diagnostic models utilizing exosomal miRNAs have predominantly been validated through ROC curve analysis (62–64), which assesses diagnostic accuracy by evaluating the specificity and sensitivity of the model. Higher specificity and sensitivity reflect an improved ability to accurately identify both positive and negative cases. Moreover, a larger AUC score signifies better overall diagnostic performance (65, 66).

### 3.2 Exosomes-derived long ncRNA

The synthesis of exosomal lncRNAs has not been fully elucidated. However, it is assumed that a considerable proportion of lncRNA transcripts may contain transposable elements (TEs), implying that they formed via TE insertion into the genetic makeup (67, 68). RNA polymerase II frequently synthesizes lncRNAs from intergenic regions, ORFs, or exonic portions of the genomic material (69). The transcription of lncRNAs typically begins at divergent promoters, which vary depending on the RNA’s directionality. Several lncRNAs are transcribed in the opposite direction from the enhancer regions of genes involved for protein synthesis. These proteins accelerate the transcription process through chromatin remodeling complexes (such as SWI/SNF) and are inhibited by CAF-1 (70, 71). The positioning of the U1 spliceosome and the 3’ UTR at bidirectional transcription sites is uniquely organized, promoting mRNA splicing in one direction while facilitating lncRNA splicing and adenine tail addition in the opposite direction. lncRNAs are distributed across various cellular regions, including chromatin, nuclear compartment, and the intracellular matrix.

lncRNAs are frequently found in various types of cancer, and their atypical expression and sequence variability are related with oncogenesis. Substantial evidence from research highlights that exosomal lncRNA us uniquely expressed in bodily fluids of various tumors. H19 lncRNA, once recognized for its tumor-suppressive role, is now associated with the activation of lung, breast, and head-and-neck cancers, as well as promoting cell growth and proliferation in bladder and hepatocellular carcinoma (HCC) (72–74). The lncRNA LINC00152 was first identified in exosomes produced by HCC in 2013. Since then, it has been shown to contribute to tumor cell adherence and proliferation (75).

Normal cells released exosomal PTENP1, which was transferred to breast cancer cells and suppressed malignant growth. It has been shown to have significantly lower levels in breast cancer tissues, demonstrating strong potential to distinguish individuals with breast cancer from normal controls, with an AUC score of 0.744 (76). Exosomal NEAT1 has been shown to contribute to the tumorigenic characteristics of gastric cancer (GC) in both *in vivo* and *in vitro* studies, not only by suppressing p53 through UBE3C and RAD18, but also by downregulating the tumor suppressor protein TP53INP1, thereby stimulating epithelial-mesenchymal transition (EMT) (77). Exosomal LINC01133 is strongly associated with increased tumor size and metastatic behavior, establishing it a viable diagnostic tool of GC (78). Serum exosome-associated lncRNA NNT-AS1 has been identified as a driver of oncogenesis in CRC via the miR-496/RAP2C signaling pathway. It also shows potential as a biomarker, with an Area Undercurve (AUC) of 0.7908 for recognizing CRC patients and healthy individuals (79). TTN-AS1 serum exosomal lncRNA were over expressed in biliary carcinoma patients, and this overexpression was implicated with the TNM and LNM stage of biliary carcinoma patients (80, 81).

MALAT1 lncRNA was considerably elevated in plasma exosomes, showing an AUC of 0.701, distinguishing NSCLC patients from control. Additionally, MALAT1 was found to have a strong association with TNM stage and lymph node invasion (82). MALAT1 has also been designated as the first exosomal lncRNA marker for Wilms’ tumor, a rare tumor of the kidney, showing lower expression in urine and plasma-derived exosomes (83). Using a multivariable logistic regression model, a panel of three lncRNAs consisting of SNHG16, UBC1, and PCAT1 was designed. The panel revealed diagnostic effectiveness for BC with substantially increased AUC values, attaining a value of 0.856 in the training dataset and 0.827 in the confirmatory dataset, showing greater accuracy than Urinary cytopathology (84–86). A four-lncRNA panel (POU3F3, UCA1, PEG10, and ESCCAL-1) within exosomes has been used for diagnosing esophageal squamous cell carcinoma (ESCC). This panel demonstrated strong efficacy, achieving an AUC score of 0.852 in the confirmatory phase. It has also proven effective in differentiating between disease stages. Kaplan-Meier analysis showed that higher levels of POU3F3 and UCA1 correlate with decreased survival outcomes (87–89). Moreover, Linc-POU3F3 may be a distinct prognostic maker for ESCC patients, with a significant p-value of 0.005 (90).

In an investigation comprising over 200 people, serum exosomal FOXD2-AS1, XLOC-009459, and NRIR levels were considerably elevated in CRC patients. The overall AUC score for all CRC patients was 0.736, while the score for early-stage CRC patients was 0.758, indicating that these markers can be effectively used for stage- specific assessment (91). A panel of four lncRNAs—GACAT2 (HMlincRNA717), GHSROS, HOTAIR, and TP53COR1 (lincRNA-p21)—achieving an outstanding AUC score of 0.937 in differentiating NSCLC from normal controls, highlighting its exceptional diagnostic potential (92, 93). A machine learning algorithms-based panel comprising twenty exosomal lncRNAs was created and examined for OC detection, indicating upregulation of exosomal lncRNAs associated with a poorer overall survival outcome (94). Exosomal-derived lncRNA-GC1 successfully differentiated GC patients from normal individuals, gastric ulcer patients, and those with enteric epithelial metaplasia, with AUC scores of 0.8861, 0.8682, and 0.8735, respectively. In comparison, conventional markers such as CA 72-4, CEA, and CA19–9 showed AUC scores below 0.8 in all tests. Notably, exosomal GC1 demonstrated an AUC score of 0.9022 in GC patients with negative CEA, CA 72-4, and CA19-9, highlighting its potential for early and effective screening of GC (95–97).

Exosomal lncRNA GAS5 plays a significant role in tumor suppression, and its reduced expression in NSCLC cells may result in activation of oncogenic pathways. Among a sample of 104 patients, GAS5 demonstrated strong diagnostic performance in differentiating NSCLC from normal controls, achieving an AUC score of 0.919 when combined with CEA. It is important to note that reduced levels of GAS5 are also associated with cancer development and advanced TNM stages. Exosomal lncRNA RP5-977B1 also has demonstrated significant diagnostic potential by effectively distinguishing NSCLC from both normal controls and lung tuberculosis, outperforming the traditional marker CEA (98). In the initial phase, a higher concentration of the unique exosomal lncRNA PEG10 was identified in NSCLC patients compared to controls, efficiently distinguishing early-phase (I & II) NSCLC cases from the reference group with an AUC score of 0.8650 (88, 99).

LncRNA CEBPA-dT (formerly CEBPA-AS1 or LOC80054) is significantly elevated in GC cells, and its presence in serum exosomes is associated with the cancer stage (TNM), increasing with more aggressive carcinomas. CeBPA-dT had a higher AUC score of 0.723 compared to convention GC markers including CA-125, ca 72-4, and CEA (100). Exosomal CRNDE was significantly associated with LNM, metastatic status, and survival outcomes. It demonstrated strong diagnostic performance in distinguishing CRC patients from individuals with non-invasive infections and healthy controls, achieving an AUC score of 0.791, sensitivity of 71.4%, and specificity of 93.3%. In contrast, CEA analysis revealed an inferior AUC score of 0.689, with sensitivity and specificity of 38.15% and 87.16%, respectively (101). Lnc-GNAQ-6:1 has been shown to be downregulated in gastric cancers and could serve as a potential target marker for GC screening, achieving AUC greater than the standard CEA and CA 72–4 markers (102). Characterization of exosomal ncRNA led to the identification of lncUEGC1, which achieved an AUC score of 0.876 for plasma-derived exosomes, effectively distinguishing stage I and stage II gastric cancer (GC) patients from normal individuals. This performance surpassed that of serum CEA, which had an AUC of 0.6614. This method effectively differentiates stage I GC patients from both normal controls and persistent atrophic gastritis patients, as well as from those with chronic disease in the first stage of GC (103). Increased expression of ENST00000457302.2 and LINC00635 in hepatocellular carcinoma (HCC) is associated with lymph node metastasis (LNM), TNM stage, and overall survival (OS). Their concentrations significantly decrease after surgical procedures, suggesting their potential utility in monitoring disease progression or recurrence. Additionally, these exosomal lncRNAs shows considerable effectiveness in differentiating HCC from persistent hepatitis B, achieving an AUC score of 0.794 when combined with plasma Alpha Fetoprotein (AFP) (104). It was found that the lncRNAs COPB2-DT (ENST00000457302.1) and ENST00000440688.1 were overexpressed in HCC patients compared to the healthy controls (HC) and chronic hepatitis (CH) groups, highlighting their potential as biomarkers for HCC diagnosis and progression (105). When combined with AFP, these lncRNAs efficiently differentiated HCC patients from both chronic hepatitis (CH) and healthy controls (HC), achieving AUC scores of 0.906 and 0.878, respectively. Furthermore, the three-lncRNA group, when paired with AFP, demonstrated strong predictive capacity for HCC invasion, with an AUC score of 0.871. The combination of lncRNA THEMIS2-211, and LINC02418 results in a high AUC score of 0.877 (106). Exosomal LINC-PINT outperforms AFP in efficiently diagnosing patients at an early stage, especially stage I patients (107). Exosomal HOTTIP expression was found to be higher in Gastric Cancer (GC) patients, with a significant correlation to the extent of invasion and TNM stage. Furthermore, elevated exosomal HOTTIP levels were associated with suboptimal overall survival, and its upregulation has been identified as a distinct risk factor in GC patients. Moreover, HOTTIP shows potential as a marker for GC, serving as both a screening and predictive tool (103).

Studies have demonstrated that lncRNAs isolated from urinary exosomes serves as an effective marker for detecting bladder cancer, indicating their potential for use in non-invasive diagnostic methods. Individuals with bladder cancer show upregulation of urinary exosomal lncRNA SNHG16. Interestingly, lncRNA SNHG16 demonstrated superior diagnostic precision with an AUC score of 0.792, substantially surpassing the standard approach of urine cytopathology (108). TALAM1, lncRNA- FAL1, TTN-AS1, and UCA1 are additional urine-derived exosomal lncRNAs that distinguish cancer patients from normal controls. In addition, a panel consisting of the four lncRNAs and nuclear mitosis related proteins were created, showing strong prognostic ability with an AUC score of 0.851 (109). Significant relationships were also discovered between tumor severity and the lncRNAs UCA1 and MKLN1-AS level. Prostate cancer patients with upregulation of PC-derived exosomal lncRNA FGD5-AS1d are associated with poor prognosis and have been shown to activate M2-type macrophage activation via the NF-kB/STAT3 signaling, leading to malignant behavior (110). This discovery paves the way for new opportunities in cancer detection and surveillance, offering less invasive diagnostic approaches with the potential to improve patient outcomes through timely interventions.

### 3.3 Exosomes-derived circular RNAs

CircRNAs represent a unique class of ncRNAs, distinct from the more common linear structures found in most ncRNA species. They form covalently closed, continuous loops, lacking the typical 5’ to 3’ polarity and poly(A) tails. They are synthesized through alternative splicing pathways, specifically via head-to-tail back splicing. Their Production is regulated by elements that function both locally (cis-acting) and form a distance (trans-acting). Complementary pairing sequences, including Alu motif, intronic complementary sequences (ICSs), reverse complementary matches (RCMs), are frequently present within the Intronic flanking sequences of circularizable exons. Circularization of exons can be facilitated by introns containing reverse complementary repeat sequences that are shorter than 100 nucleotides (111). It plays pivotal role in regulating gene expression by influencing both gene transcription and post-transcriptional processes. CircRNAs show enhanced resistance to RNase degradation and greater stability compared to linear RNA transcripts. This distinctive feature makes them highly valuable as markers for cancer detection.

From a functional perspective, circRNAs play diverse roles in cancer biology, influencing tumorigenesis through various pathways. Some circRNAs act as oncogenes, promoting oncogenesis and tumor progression. For instance, CircHIPK3 has been implicated in the pathogenesis of intrahepatic cholangiocarcinoma (iCCA) and breast cancer (BC), where it contributes to tumor growth and metastasis (112). In contrast, other circRNAs function as tumor suppressors. For example, circMTO1 has been shown to inhibit tumor progression in hepatocellular carcinoma, while circFNDC3B plays a suppressive role in bladder cancer (113). These examples highlight the complex and multifaceted roles circRNAs play in cancer biology, influencing both tumor development and suppression by regulating key cellular pathways. Further evidence supporting the potential of circRNAs in personalized oncology is seen in their differential expression across various cancers. For example, circ-0001821 is downregulated in GC tissues but upregulated in CRC tissues (114). This unique expression pattern suggests that circ-0001821 could be a promising diagnostic marker or therapeutic target, underscoring the potential f circRNAs in personalized cancer treatments.

Research has shown that circRNAs are abundant and stable in exosomes, as confirmed through qRT-PCR analysis of tumor-associated tissues. Notably, the levels of circ-KLDHC10 were significantly higher in the plasma of CRC-positive patients compared to normal controls (115). Additionally, malignant brain tumor associated microglia-secreted exosomal circKIF18A influences nuclear trafficking in human brain microvascular endothelial cells (HBMECs) and enhance angiogenesis in Glioblastoma (116). Serum exosomal circRNA-100284, upregulated in cisplatin-resistant lung carcinoma cells, sponges miR-122 and increases HK2 function to stimulate glycolytic activity and cancer progression. Combining si-circ-0008928 with 2-DG may optimize the therapeutic responses. Exosomal ciRS-122 from oxaliplatin-adaptive CRC cells may be transferred to susceptible cells, where it targets miR-488 and upregulates PKM2, enhancing glycolysis and contributing to drug resistance. Likewise, circFOXK2 stimulates the Pyruvate Dehydrogenase Complex (PDC) pathway and influences the miR-484/Fis1 axis in HCC, contributing to mitochondrial fragmentation, oxidative glycolysis, and pulmonary metastasis (117). The Warburg effect drives chemotherapy-resistant glioma cells to release exosomal circ-0072083, thereby enhancing resistance to Temozolomide.

Research has shown that CircHIPK3 is significantly overexpressed in a wide variety of cancers, including those of the kidneys, gastrointestinal tract, liver, lungs, gallbladder, pancreas, cervix, and ovaries. This widespread overexpression suggests that CircHIPK3 plays a crucial role in oncogenesis across different tissues. Furthermore, RNAi-mediated silencing of CircHIPK3 has been shown to effectively suppress cellular growth in CRC, highlighting its potential role in regulating cellular development and its promise as a therapeutic target. Further investigations have revealed that CircHIPK3 acts as a sponge for miRNAs, influencing various aspects of tumor growth, including heterogeneity, proliferation, invasiveness, and metastasis. Additionally, CircHIPK3 was found to be downregulated in bone cancer, correlating with decreased overall survival. Its expression levels were also linked to the progression of musculoskeletal tumors, as indicated by the Enneking stage, and to pulmonary involvement. ROC analysis indicated that CircHIPK3 could be a potential diagnostic marker for bone cancer, with an AUC score of 0.784 (118, 119). CircSHKBP1 promotes GC development by elevating HUR and VEGF expression through the suppression of HSP90 and the sponging of miR-582-3p, thereby disrupting STUB’s activity (117). Exosomal circ-0000735 was shown to be overexpressed in patients with NSCLC, facilitating tumorigenesis and metastatic progression by sequestering miR-21 and targeting ADAM19 (120). Overexpression of plasma exosomal circ-ATP8A1 in GC patients has been linked to cancer Immune evasion, advanced tumor stage, metastasis, poor clinical outcome. circ-ATP8A1 also trigger M2 macrophage polarization via circATP8A1–miR-1-3p–STAT6 cascade, thereby enhancing GC migration (121).

While individual circRNAs have proven reliable for cancer detection, sets of circRNA markers have demonstrated even greater potential in enhancing diagnostic accuracy and outcomes. A panel of two circRNAs (circ-0058124 and circ-RAPGEF5) were used to diagnose individuals with papillary thyroid cancer (PTC). To evaluate the clinical utility of this group in greater detail for discriminating between PTC and non-malignant individuals, the results demonstrated its effectiveness in distinguishing PTC from lymphadenopathy, goiter, and neck lumps. The group attained an AUC score of 0.806, with sensitivity and specificity of 81.2% and 64.1%, respectively, for PTC (122). A set of three circRNAs, comprising circABCC2, circCCDC66, and circPVT1, exhibited substantial downregulation in CRC patients. The findings also suggest that circABCC2 and circPVT1 are useful for identifying dysplasia, non-cancerous hyperkeratosis, surgical intervention, and grading CRC (123, 124). A Panel of serum circRNA (circ-CDR1as and circCCDC66) demonstrated greater diagnostic accuracy for AFP-tive HCC and AFP-tive early-phase HCC. The panel also exhibited an AUC score of 0.763 for HCC, whereas the AUC score for AFP was 0.791. When integrated two circRNA marker dataset with AFP, the AUC score can be raised to 0.864. For diagnosing small HCC, the circRNA dataset exhibited an AUC score of 0.861, whereas the integrated panel achieved an AUC score of 0.874 (125).

The upregulation of circ-0034398 in esophageal squamous cell carcinoma (ESCC) was found to be associated with anaplastic features, tumor staging, and TNM score, as reported by (124). Their findings also showed that patients with high-grade, poorly differentiated tumors had higher levels of circ-0067934. Additionally, patients with early-stage TNM (I-II) cancer exhibited higher circ-0067934 expression compared to those with advanced stages (III-IV) (126). CircRNA-100290 was found to be suppressed in GC tissues, expression patterns were markedly correlated tumor staging, metastatic spread, gender, and age group in BC. CircRNA-100290 has an AUC score of 0.729, 70% specificity, and 70% sensitivity, rendering it a viable marker for BC detection.

Depending on the presence or absence of target molecules such as androgen receptors, PGRMC1, and the PI3K/AKT/mTOR pathway, breast cancer is most commonly classified into three categories: estrogen receptor (ER)-positive/ERBB2-positive, ERBB2-negative, and triple-negative breast cancer. Few tumor-associated circRNAs were frequently observed in BC subgroups, while others exhibited distinct molecular signatures. The studies indicated that the ER-stimulated BC subgroups have a greater number of circRNAs in the paraneoplastic lesions compared to oncogenic tissues (126). These findings highlight that circRNAs exhibits exceptional selectivity, sensitivity, and reliability, making them as promising candidates for early cancer detection.

### 3.4 Other exosomal ncRNAs

Some exosomal ncRNAs, along with the well-known miRNAs, lncRNAs, and circRNAs, also have significant impacts on tumor characteristics. For example, tRNA-derived fragments (tRFs) are a subset of ncRNAs formed when endoribonucleases cleave tRNA precursors. Previous studies suggest that tRFs may act as post-transcriptional modulators, functioning similarly to miRNAs or interacting with RNA-binding proteins. tRFs also influence translational activity by inhibiting ribosome biogenesis and initiation, thereby impacting the overall efficiency of protein synthesis. tRFs have been identified as aberrantly expressed in tumor tissues, playing a crucial role in cancer development, metastasis, invasion, and neovascularization through various signaling pathways. As a result, these extracellular tRFs are considered promising markers for cancer diagnosis and outcome prediction, particularly in liquid biopsy-based evaluations (127).

The saliva of patients with ESCC exhibited an overexpression of exosomal 5’-tRNA-GlyGCC and an unidentified ncRNA sRESE, both of which were linked to metastasis, migration, and cell proliferation. The AUC score for the two ncRNAs were 0.877 and 0.872, respectively, and 0.934 when used in combination, demonstrating their viability for ESCC diagnostics (87, 128). Research has shown that plasma exosomal levels of tRNA-GluCTC-5, tRNA-GlyTCC-5, and tRNA-ValTac-3 were substantially increase in patients with hepatic cancer. This highlights the potential of circulating exosomal tsRNA as a valuable marker for the preliminary screening of hepatic cancer, distinguishing it from non-benign conditions such as cirrhosis, primary sclerosing cholangitis (PSC), and drug-induced liver injury (DILI), offering a significant opportunity for improved diagnosis and patient outcomes (129).

It has been observed that Ser-TGA-001, tRF-Gly-CCC-008, tRF-Glu-CTC-003, tRF-Ser-TGA-002, and tRF-Leu-CAA-003 were found to be markedly suppressed in plasma isolates of patients with pre-invasive BC (130, 131). In another study, NSCLC patients exhibited significantly lower levels of tRF-Lys-CTT-049, tRF-Leu-TAA-005, tRF-Trp-CCA-057, and tRF-Ala-AGC-036 in plasma exosomes compared to non-exosomal supernatants. Downregulation of exosomal tRF-Ala-AGC-036 is closely linked to the T/N stage, providing a crucial means to differentiate between patients with preliminary stage and those with late-stage NSCLC (132).

The differential expression of tRFs in early and late-stage cancers, such as NSCLC, highlights their potential utility in disease stratification and monitoring progression. These findings pave the way for developing multi-marker sets, such as bi-ncRNA combinations, for more precise and personalized cancer diagnostics. Collectively, these insights establish exosomal tRNAs and tsRFs as powerful tools for advancing liquid biopsy-based approaches, offering a minimally invasive, highly accurate, and practical avenue for cancer management and patient care. A comprehensive list of available exosomal ncRNAs is presented in **Table 1**.

**Table 1:** List of exosomal ncRNAs, including details on their sources, methods for diagnostic assessment, corresponding tumor types, expression patterns, area under curve, and source study.

Exosomal ncRNA	Source	Diagnostic methods	Tumor type	Expression	Characteristics	AUC score	Reference
Exosomal miRNA							
miR-92a, miR-150, and miR-21	Saliva	RT-qPCR (n=20)	HNSCC	Increased	Immune modulation, Cellular signaling	NA	(133)
miR- 200b-3p, miR- 429, miR-1229, miR-216b-5p, miR-1246, and miR-25-3p	Plasma	RT-qPCR (n = 192)	PDAC	Increased	EMT, Cancer signaling pathways	AUC were 0.92, and 0.99 in combination with CA19-9	(134)
miR- 200c, miR-200b, , and miR-222	Pleural exudate	RT-qPCR (n=36)	LUAD	Increased	NA	AUC 0.95	(135)
miR-4732-5p	Plasma	RT-qPCR (n=54), ddPCR	EOC	Stage specifically Increased	NA	AUC 0.889	(136)
miR-30a-5p, miR-216b-5p, miR-150-5p, miR-26b- 5p	Serum	NA	CRC	Increased, decrease from stage II and IV CRC	Cancer metabolism, Proteoglycan	NA	(137)
miR-92b-5p	Serum	RT-qPCR (n=113)	BC	Increased	Cell invasion, Focal adherence, Survival rate	AUC 0.786, 0.673, 0.873, and 0.792, respectively, for overall and stages I and III of BC	(138)
miR-34-5p, and miR-193a-3p	Serum	RT-qPCR (n=275)	NSCLC (0 and I stage)	Decreased	NA	AUC 0.811 and 0.672, respectively, 0.848 in combination; 0.827 and 0.921 in combination with CEA and CYFRA21-1.	(139)
miR-382	Serum	RT-qPCR (n=185)	NSCLC	Downregulated	Lymphatic invasion, survival outcomes	AUC 0.901	(140)
miR-205	Plasma	RT-qPCR (n=98), ddPCR	OC	Increased	Lymphatic proliferation, survival outcomes	AUC 0.952	(141)
Exosomal lncRNAs							
PTENP1	Plasma	RT-qPCR (n=110)	BC	Decreased	Tumor invasion, Metastasis, Autophagy	AUC 0.742	(76)
MALAT1	Serum	RT-qPCR (n=107)	NSCLC	Increased	Tumor invasion, Metastasis, Autophagy	AUC 0.704	(142)
POU3F3, PEG10, and LINC02418,	Serum	RT-qPCR (n=221)	ESCC	Increased	Metastasis, Cytotoxicity, G1/S phase arrest,	AUC 0.843, 0.706, 0.655, and 0.650, respectively, with 0.854 in combination; AUCs were 0.821 for tumor classification I, II and 0.934 for stage III.	(143)
lncRNA- FAL1	Serum	RT-qPCR (n=825)	HCC	Increased	Cancer staging	AUC 0.904	(144)
PCAT1, and SNHG16	Serum	RT-qPCR (n=320), ddPCR	BC	Increased	Invasion, extravasation, and Lymphatic metastasis	AUC 0.826	(145)
CEBPA-AS1	Serum	RT-qPCR (n=104)	NSCLC	Increased	Clinical staging, Tumor volume	AUC 0.928, 0.821 for Stage 1 lung cancer	(146)
lncUEGC1	Plasma	RT-qPCR (n=111)	Stage I, II GC	Increased	NA	AUC 0.776, AUC 0.851 for stage I-II of GC	(147)
HOTTIP	Plasma	RT-qPCR (n=246)	GC	Increased	Clinical staging, Tumor volume	AUC 0.828, 0.871, with combination with CEA, CA72–4	(148)
FGD5-AS1	Tumor sample	RT-qPCR (n=240)	PC	Increased	Promote M2 macrophage activation	NA	(149)
SOX2-OT	Plasma	RT-qPCR (n=200), ddPCR	LSCC	Increased	MKLN1-AS and UCA1 tumor staging, overall survival	AUC 0.807	(150)
Exosomal circRNAs							
circACTN4	Tumor sample	RT-qPCR (n=77)	ICC	Increased	Hyperplasia, Invasive potential, Clinical outcome	NA	(151)
circKLDHC10	Serum	RT-qPCR (n=11)	CRC	Increased	NA*	NA	(152)
circATP8A1	Plasma	RT-qPCR (n=142), ddPCR	GC	Increased	Clinical staging, Tumor volume	NA	(153)
circHIPK3	Tumor sample	RT-qPCR (n=82)	OC	Decreased	Chemotaxis, Infiltration, Clinical outcomes	AUC 0.783	(154)
circ-0044516, and circ-0000976	Plasma	RT-qPCR (n=1195)	HCC	Increased	TNM stage, Invasion, extravasation, and Lymphatic metastasis	AUC 0.844 and 0.865 in trail, Clinical staging, Tumor volume	(155)
circSTIL, and circABCC1	Plasma	RT-qPCR (n=210), ddPCR	CRC	Decreased	Hyperplasia, Invasive potential, Clinical outcome	AUC 0.755	(156)
circRUNX1	Tumor sample	RT-qPCR (n=54)	ESCC	Increased	TNM stage, Migration, invasion, metastasis,	AUC 0.893	(157)
Circ-0034398, and circRAPGEF5	Serum	RT-qPCR (n=304)	PTC	Increased	Tumor growth, angiogenesis, metastasis	AUC 0.691 and 0.726 respectively, 0.866 in combination	(158)
circLPAR1	Plasma	RT-qPCR (n=200), ddPCR	CRC	Decreased	TNM stage, Tumor volume	AUC 0.732	(159)
Circ-0067934	Tumor sample	RT-qPCR (n=137)	ESCC	Increased	TNM stage, Invasion, extravasation, and Lymphatic metastasis	NA	(160)

## 4 Clinical application, and challenges

### 4.1 Clinical applications

**Figure 3:**
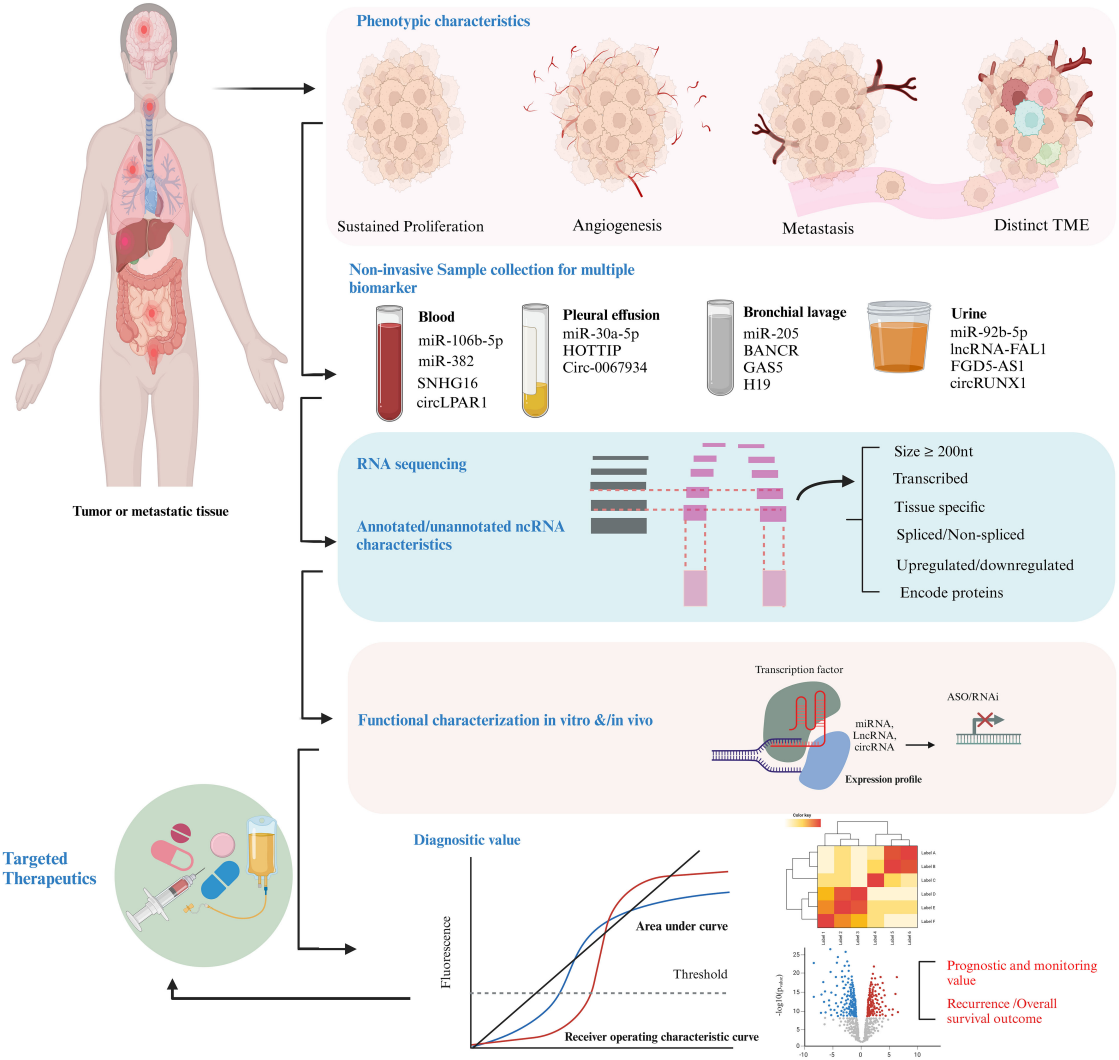
Methodological framework for investigating ncRNAs as markers and therapeutic targets in cancer. Initially, hallmark phenotypic characteristics, including sustained proliferative signaling, angiogenesis, and metastatic potential, are evaluated. ncRNA cargoes, such as miRNAs, lncRNAs, circRNAs, and tsRFs are isolated from EVs present in various body fluids using liquid biopsy techniques. qPCR, qRT-PCR is employed for transcriptomic profiling, followed by functional characterization of ncRNAs by siRNA/shRNA Knockdown, RNA-seq/Microarrays and CRISPR/Cas9. The diagnostic utility is evaluated through metrics such as AUC scores and ROC curve analysis to determine sensitivity and specificity, alongside assessments of recurrence rates and overall survival (OS) outcomes. Finally, ncRNAs with established roles are prioritized for therapeutic interventions through strategies such as antisense oligonucleotides (ASOs) and RNA-based therapeutics.

For many reasons, exosomal ncRNAs hold exceptional capability as diagnostic and prognostic markers in cancer. Firstly, numerous investigations have shown that exosomal ncRNAs are often dysregulated in various types of malignancies. Secondly, they reflect the molecular signatures of the cells from which they are derived. Thirdly, they have ability to represent the molecular contents and genetic alterations present not only in the primary tumor but also in metastatic sites. Currently, they are being explored to provide real-time insights into tumor progression. For example, lncRNA PC antigen-3 (PCA-3), approved by the FDA in 2012 as the PROGENSA PCA-3 assay, serves as a urine-based marker for Prostate Cancer (PC) by measuring the ratio of PCA3 to prostate-specific antigen (PSA) levels (161). Exosomal PCA-3 showed significant upregulation following a physical rectal evaluation, with diagnostic effectiveness surpassing that of PCA-3 obtained from cellular pellets, highlighting its potential as an exceptionally reliable marker (162). Additionally, exosomal lncUEGC1 demonstrates superior diagnostic value compared to conventional serum CEA in early-stage gastric cancer patients. While PCA3 and lncUEGC1 have successfully transitioned into clinical application due to their robust validation, ease of implementation, and regulatory approval, exosomal ncRNAs face substantial challenges, including regulatory uncertainty, validation bottlenecks, and significant barriers to real-world integration. Addressing these obstacles will require the development of standardized protocols, further comprehensive clinical trials to solidify their clinical utility, and the establishment of clear regulatory frameworks to enable the widespread adoption of exosomal ncRNAs as reliable cancer biomarkers, as summarized in **Table 2**. Additional characteristics, such as the high stability of exosomal ncRNAs in bodily fluids, further enhance their reliability as markers. Enclosed within the protective lipid bilayer of exosomes, they are shielded from enzymatic degradation, allowing them to endure cryogenic temperature shifts and thermal robustness at −25°C for up to five years. This durability ensures that exosomes can serve as a consistent and dependable source of ncRNAs, enabling long-term preservation and recovery for diagnostic applications. **Figure 3** illustrate a comprehensive approach to target ncRNAs as both cancer biomarkers therapeutic agents.

**Table 2:** Clinical research exploring the diagnostic significance of exosomes derived ncRNAs.

Clinical Trail	Research status	Tumor type	ID Number
Exosome-derived liquid Biopsy for Primary liver cancer (ELUCIDATE)	Recruiting since 2024	HCC	NCT06342414
Colorectal Cancer diagnosis in early stage (ENCODE)	Recruiting since 2024	CRC	NCT06342401
Exosomal miRNA Profiling for Evaluating Prostate Cancer Aggression	Completed 2021	PC	NCT03911999
Adenomatous Polyps and Colorectal Cancer (AACRC)	Recruiting since 2024	AA/CRC	NCT06342440
Exosomal miRNA in serum for Therapeutical interventions	Recruiting since 2024	SCC	NCT05854030
LNM diagnosis in Intrahepatic Cholangiocarcinoma (LyMIC)	Recruiting since 2024	ICC	NCT06381648
Serum exosomal lncRNAs as Potential markers for pulmonary Tumor characterization	Completed 2021	LC	NCT03830619

### 4.2 Challenges

Exosomal ncRNAs face several significant challenges that hinder their clinical application, particularly in the areas of biology, technical execution, and regulation. Biologically, the intricate roles of exosomal ncRNAs in various cellular pathways complicate their functional evaluation. The differential expression of these ncRNAs across different organs further reduces their specificity as reliable diagnostic markers. For instance, exosomal lncRNA SNHG15 is elevated in multiple tumor types, but its expression is also observed in normal cells, reducing its diagnostic exclusivity. Similarly, miR-425-5p exhibits higher expression in PDAC patients compared to adjacent tissues, but its predictive value varies across different cancer types, complicating its role in cancer diagnostics. Additional variability is seen with miR-20b-5p, which is downregulated in early NSCLC but upregulated in prostate cancer patients’ ejaculate. This kind of contextual variability, along with the inherent heterogeneity of tumors and the lack of symptoms before metastasis, further complicates the accurate early diagnosis of cancer.

Technically, a major challenge in utilizing exosomal ncRNAs as diagnostic markers lies in the limited understanding of how ncRNAs are packaged into exosomes. The mechanisms behind the selective packaging of ncRNAs into exosomes remain poorly understood, making it difficult to standardize and reproduce results for diagnostic purposes. Furthermore, while exosomes containing ncRNAs are often tested in controlled environments, it remains uncertain whether the levels of ncRNAs in these exosomes reflect actual physiological conditions *in vivo*. Another critical technical barrier is the small sample sizes commonly used in many studies, which restrict the generalizability of the findings and hinder the establishment of reliable diagnostic thresholds. Additionally, some exosomal ncRNAs show limited diagnostic effectiveness due to low sensitivity and specificity. For example, the sensitivity of saliva-derived exosomal miR-517a-3p and miR-486-3p in detecting head and neck squamous cell carcinoma (HNSCC) was found to be only 18% and 44%, respectively, severely limiting their practical utility in diagnostics.

Regulatory challenges also pose significant obstacles to the clinical use of exosomal ncRNAs. The absence of standardized methods for isolating exosomes and profiling ncRNAs leads to variability in exosome populations, which affects the reliability and reproducibility of results. Moreover, there are currently no universally accepted guidelines for the normalization of exosomal ncRNA data, complicating cross-study comparisons and validation efforts. The regulatory framework for exosome-based diagnostic assays is still unclear, with no established criteria for assay validation or clinical utility. This regulatory uncertainty slows down the approval process and impedes the adoption of exosomal ncRNAs as clinically validated diagnostic markers. Therefore, there is a pressing need for clear regulatory guidelines and standardized protocols to facilitate the integration of these biomarkers into clinical practice.

## 5 Conclusion

Exosomal ncRNAs hold promising potential as diagnostic and prognostic markers for cancer, thanks to their unique properties, such as stability, specificity, and ability to reflect the molecular characteristics of primary and metastatic cancers. Their encapsulation in exosomes protects them from enzymatic degradation, making them a valuable resource for liquid biopsy-based diagnostics. However, several challenges must be addressed before their clinical integration. The intricate roles of exosomal ncRNAs in cellular pathways, along with their differential expression across various organs, complicate both their functional evaluation and diagnostic utility. To overcome these obstacles, there are key next steps required. Clinical standardization is essential, including the development of standardized protocols for exosome isolation, ncRNA profiling, and data normalization to ensure consistency and reproducibility across studies and clinical settings. Additionally, deeper functional studies are necessary to understand the specific roles of exosomal ncRNAs in cancer progression and their differential expression across different cancer types and tissues, improving their specificity and accuracy as biomarkers. Lastly, bioinformatics integration will play a critical role by applying advanced data analysis tools to integrate multi-omics information, improving the identification and validation of exosomal ncRNA signatures. By addressing these challenges, exosomal ncRNAs have the potential to revolutionize cancer diagnostics, enabling early detection and personalized treatment through non-invasive and highly accurate biomarker systems.
